# Bladder and rectal incontinence without paraplegia or paraparesis after endovascular aneurysm repair

**DOI:** 10.1186/s40792-016-0140-y

**Published:** 2016-02-11

**Authors:** Naritomo Nishioka, Yoshihiko Kurimoto, Ryushi Maruyama, Kosuke Ujihira, Yutaka Iba, Eiichiro Hatta, Akira Yamada, Katsuhiko Nakanishi

**Affiliations:** Department of Cardiovascular Surgery, Teine Keijinkai Hospital, 12-1-40, Maeda 1-jo, Teine-ku, Sapporo, Hokkaido 006-8555 Japan

**Keywords:** Bladder and rectal incontinence, Bladder and rectal complications, Abdominal aortic aneurysm (AAA), Endovascular aneurysm repair (EVAR), Aorto-uniiliac (AUI) device, Hyperbaric oxygen (HBO) therapy

## Abstract

Spinal cord ischemia is a well-known potential complication of endovascular aneurysm repair (EVAR), and it is usually manifested by paraplegia or paraparesis. We describe a case in which spinal cord ischemia after EVAR presented by isolated bladder and rectal incontinence without other neurological deficits. A 63-year-old woman presented with intermittent claudication secondary to an infrarenal abdominal aortic aneurysm (AAA), and a left common iliac artery obstruction, for which she underwent EVAR using an aorto-uniiliac (AUI) device and ilio-femoral artery bypass. On postoperative day 3, she developed urinary and fecal incontinence without signs of paraplegia or paraparesis. Magnetic resonance imaging (MRI) showed a hyper-intense signal in the spinal cord. She received hyperbaric oxygen (HBO) therapy and was discharged after 18 days when her urinary and fecal incontinence were almost resolved. This report suggests that spinal cord ischemia after EVAR for aortoiliac occlusive disease might present as bladder and rectal incontinence without other neurological manifestations.

## Background

Neurologic complications due to spinal cord ischemia (SCI) after elective infrarenal abdominal aortic aneurysm (AAA) repair, while catastrophic, rarely happen. Approximately 1 in 400 cases occur after open AAA repair while 1 in 5000 occur after arterial reconstruction of occlusive aortoiliac disease [1]. Possible causes of these adverse events include infarction of the spinal cord from direct interruption of the cord blood supply or a critical collateral, systemic hypo-perfusion, atheromatous embolic infarction, or spontaneous thrombosis of an atherosclerotic radicular artery [2]. Prolonged aortic occlusion, intraoperative hypotension, and interruption of the internal iliac artery circulation have also been proposed as possible causative factors [3]. Perfusion of the distal spinal cord is derived from the lumbar, iliolumbar, and lateral sacral arteries. Branches of these arteries anastomose with the intrinsic spinal arteries at the level of the conus medullaris. When the greater radicular artery is compromised, however, pelvic blood supply can become critically important.

We describe a case of a woman who developed isolated bladder and rectal incontinence without paraparesis/paraplegia after endovascular aneurysm repair, treated with hyperbaric oxygen (HBO) therapy, and with significant clinical improvement after 3 weeks.

## Case presentation

**Fig. 1:**
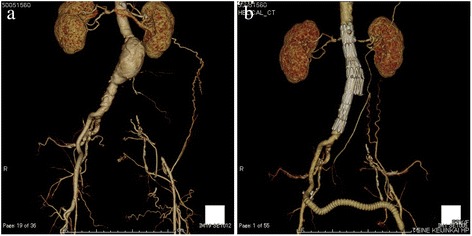
Computed tomography shows preoperative (**a**) and postoperative (**b**) conditions. Both internal iliac arteries are preserved

**Fig. 2:**
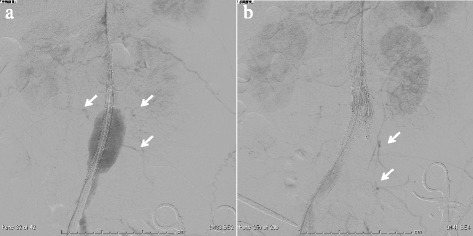
Intraoperative angiogram shows that a few large lumbar arteries (*arrows*) arising from the AAA were patent but the pre-sacral artery was occluded (**a**) and the pelvic flow (*arrows*) preserved after post-EVAR (**b**)

A 63-year-old woman with a history of hypertension and chronic kidney disease presented with intermittent claudication. A computed tomography (CT) revealed a 41-mm infrarenal AAA with total obstruction of the left common iliac artery. The bilateral external and right internal iliac arteries were patent. The distal left internal iliac artery was occluded but filled through collateral circulation (Fig. 1). A few large lumbar arteries arising from the AAA were patent, but the pre-sacral artery was occluded (Fig. 2). Given the size of her aneurysm and the risk of expansion, there was indication to perform surgery for both the AAA and the occluded left common iliac artery. In general, guidelines recommend an open approach in patients younger than 65 years of age, given the availability of long-term outcomes data. Therefore, this was the approach recommended by the team; however, the patient rejected this option and favored the endovascular approach because she desired fast symptomatic improvement of her claudication.

After a thorough risk-benefit discussion with the patient, we performed an endovascular repair with an aorto-uniiliac (AUI) device (Zenith flex, Cook medical) and ilio-femoral artery bypass using an 8-mm vascular graft (Goretex ePTFE graft) in a “hybrid” operating room under general anesthesia. After bilateral groin incision to expose the right and left common femoral artery, a bifurcated stent graft (main body of 22 mm in the proximal diameter, TFFB-22-82-ZT) was placed just below the renal artery branch section to the right common iliac artery through the right external iliac artery, followed by placing a converter of 24 mm in a proximal diameter (ESC-24-12-80) and an iliac extender of 16 mm in a distal diameter (TFLE-16-56-ZT) for the right common iliac artery. An arteriogram confirmed adequately positioned endografts, with patent bilateral renal arteries without evidence of proximal or distal endovascular leak. Subsequently, the right external iliac artery was cut longitudinally for the inflow for the bypass. The right external iliac artery and an 8-mm vascular graft (Goretex ePTFE graft) were anastomosed by using a parachute technique with a CV6 needle. The left common femoral artery was also identically anastomosed to the graft (Fig. 1). The total operating time was 3 h and 19 min, and the blood loss was 468 ml; the patient tolerated the procedure well and remained hemodynamically stable throughout the operation.

**Fig. 3:**
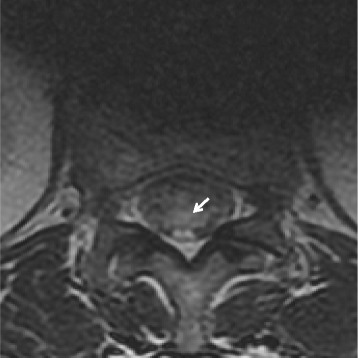
MRI shows a hyper-intense signal at the lower end of the conus medullaris in the T2-weighted image. The *arrow* points to the area of the ischemic lesion, which is white in contrast to normal nerve tissue

Seventy-two hours postoperatively, after removing the urinary catheter, she complained of urinary incontinence. A urologist and a neurologist examined the patient and agreed that there was bladder and rectal areflexia but no motor deficits. There was no livedo reticularis, and the renal function remained stable. Magnetic resonance imaging (MRI) showed a hyper-intense signal at the lower end of the conus medullaris visible in the T2-weighted image. (Fig. 3). Cerebrospinal fluid (CSF) studies were not indicated due to the late discovery of symptoms (3 days after the surgery). She underwent 10 sessions of HBO therapy at two times the atmospheric pressure for 90 min each. She was discharged on postoperative day 18 when her urinary and fecal incontinence were almost completely resolved.

### Discussion

SCI has been reported after endovascular aneurysm repair (EVAR) either as an immediate or a delayed finding [4]. An analysis of the EUROSTAR database including 2862 patients who have undergone EVAR found an incidence of SCI of 0.21 % [5]. Peppelenbosch et al., however, reported that SCI occurred in 4 out of 35 (11.5 %) patients with a ruptured AAA after the deployment of an AUI device [6]. Bajwa et al. also reported bilateral lower extremity motor and sensory loss with no bladder dysfunction after the deployment of an AUI device [7]. No reports, however, have been published about isolated bladder and rectal incontinence after EVAR.

The process leading to SCI after elective surgical management of an infrarenal AAA has not been fully understood. Factors that may contribute to it after EVAR include atheromatous embolization, interruption of the great radicular artery (artery of Adamkiewicz), or collateral circulation (internal iliac arteries-lumbar arteries) [1, 4, 7]. In our case, because lumbar arteries were “sacrificed” after the EVAR, there is a possibility that flow to the great radicular artery and the collateral circulation might have been interrupted causing this patient’s SCI.

Atheroembolization is a well-known complication of endovascular surgery and may lead to SCI. Rockman et al. reported two cases of paraplegia as a result of atheroembolization to the spinal cord after successful or attempted endovascular management of AAA [4]. In the EUROSTAR registry, factors associated with intraoperative microembolization included long procedure time (>150 min), extensive intravascular handling, and preoperative or perioperative embolization of the hypogastric and lumbar arteries [5]. In our case, the ilio-femoral artery bypass improved iliac artery flow, but SCI still occurred. Also, this patient did not have other signs of atheroembolization such as livedo reticularis or acute renal failure. The development of urinary and fecal incontinence in our patient might have been secondary to the interruption of the great radicular artery, atheroembolization, prolonged operation time, and bleeding. Balloon dilatations, deployment of the proximal converter, and distal iliac extension may have increased the risk of atheroembolization. The improvement of endovascular devices by reduction in their profile (size and length) in combination with improvement in the surgeon’s manipulation technique can be beneficial and may eventually lead to a reduction in the risk of atheroembolization in the future.

The patient’s clinical presentation is explained by the anatomy of the conus medullaris. The conus medullaris is the lower end of the spinal cord, and it usually occurs at the level of the first lumbar vertebra. The segments of spinal cord that give origin to the nerves that control the bladder and bowel sphincters are located below the third sacral segment (S3). An injury to the conus medullaris below the third sacral segment (S3) will manifest with bladder and rectal incontinence and symmetric sensory loss around the anus and sexual organs. Motor dysfunction will not develop because most of the lower extremity motor nerves originate above the S2 segment of the spinal cord [8]. Although it was unrelated to an EVAR procedure, Inatomi et al. [9] previously reported three patients with spinal cord ischemia whose symptoms started with urinary dysfunction without paraplegia or paraparesis. In our case, the clinical manifestations and imaging finding supported the diagnosis of bladder and rectal incontinence due to a spinal cord injury at the level of the conus medullaris.

The goal of the treatment for SCI is to augment spinal cord perfusion pressure and reduce edema. The usefulness of the COPS protocol (CSF drainage to maintain a pressure of less than 5 mmHg without limit under CSF drain status/oxygen delivery/patient status) has been reported after delayed neurologic deficit in thoracoabdominal aortic aneurysm repair [10]. Other therapeutic strategies include CSF drainage, steroids, arterial pressure augmentation, hypothermia, and HBO treatment. We did not perform CSF drainage since it carries a risk for further complications, and its benefit for delayed bladder and rectal incontinence is unproven.

HBO therapy has sporadically demonstrated a decrease in the size of infarcts [11]. In the above case, HBO therapy was selected because of its non-invasive nature; however, other strategies, such as CSF drainage and steroids, may be more beneficial in selected cases. Although there are no guidelines on the number and length of time of HBO treatment, the above patient underwent the standard method of two times the atmospheric pressure for 90 min.

## Conclusions

We describe a case of a woman who developed isolated bladder and rectal incontinence without paraparesis/paraplegia after EVAR, and HBO might be beneficial for improving the symptoms.

## Consent

Written informed consent was obtained from the patient for publication of this case report and any accompanying images.

## Abbreviations

AAA: abdominal aortic aneurysm
AUI: aorto-uniiliac
CSF: cerebrospinal fluid
CT: computed tomography
EVAR: endovascular aneurysm repair
HBO: hyperbaric oxygen
MRI: magnetic resonance imaging
SCI: spinal cord ischemia
